# Karyopherin Dysfunction Is a Key Driver of Aging

**DOI:** 10.1111/acel.70634

**Published:** 2026-07-13

**Authors:** Louis R. Lapierre

**Affiliations:** ^1^ New Brunswick Centre for Precision Medicine Moncton New Brunswick Canada; ^2^ Université de Moncton Moncton New Brunswick Canada

## Abstract

Aging is often framed as the gradual erosion of proteostasis, driven by declining chaperone capacity, impaired degradation, and dysregulated protein synthesis. Yet this view implicitly assumes that proteins fail primarily because they misfold or escape clearance. Increasing evidence instead points to a more fundamental problem: aging disrupts the spatial management of the proteome. Gradually, proteins are misplaced, signaling pathways are uncoupled from their compartments, and condensates that were once dynamic become pathological. At the center of this spatial collapse lies nucleocytoplasmic protein partitioning. Nucleocytoplasmic protein transport has long been treated as a background housekeeping process, that is, essential but largely passive. However, this assumption is no longer reasonable. Karyopherins, the importins, exportins and biportins that mediate selective transport across the nuclear pore complex (NPC), are emerging as active regulators of proteostasis, phase behavior, and signaling fidelity. Rather than simply responding to cargo demand, karyopherins shape intracellular protein solubility, suppress aberrant condensation, and buffer age‐associated stress. Their dysfunction therefore constitutes a primary, not secondary, driver of aging phenotypes. Here, I argue that karyopherins should be repositioned at the core of aging biology. I propose that age‐dependent failure of karyopherin‐mediated transport represents a unifying mechanism linking proteostasis collapse, altered gene regulation, and the emergence of age‐associated diseases. This perspective redefines nucleocytoplasmic protein transport from a logistics challenge into a central regulatory layer and highlights karyopherins as emerging targets for aging interventions.

## Graphical abstract

Studies on the proteostasis network and aging have focused on protein synthesis, folding, and turnover. Here, emphasis on the spatial dimension in proteostasis is proposed by recognizing nucleocytoplasmic transport as an important player in aging.

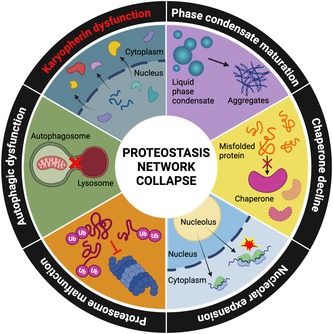

## 1 Spatial Proteostasis: A Missing Dimension of Aging Biology

**FIGURE 1:**
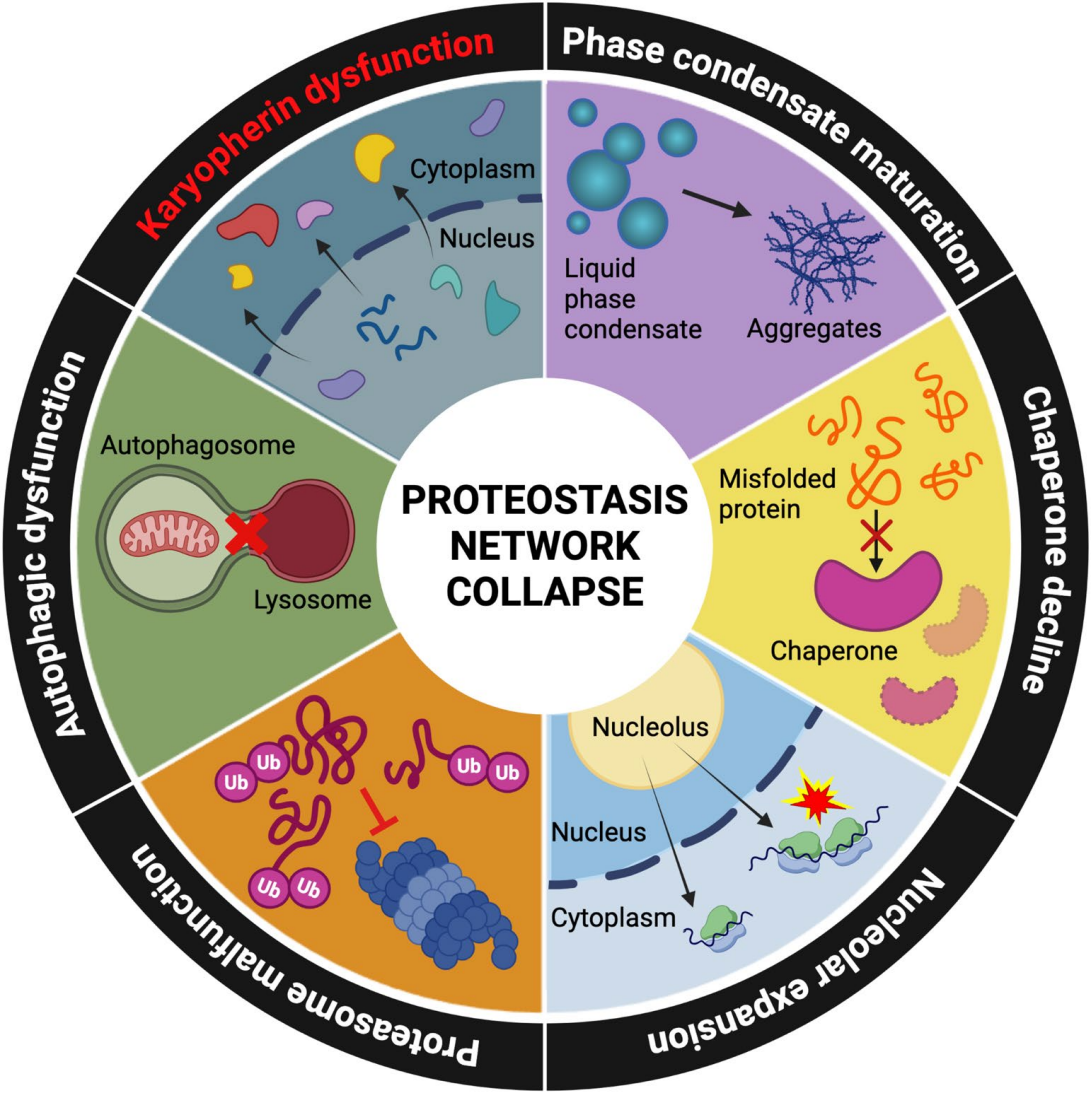
Proteostasis depends on nucleocytoplasmic protein partitioning. The integrity of the proteostasis network relies on the precise and dynamic spatial distribution of proteins between the nucleus and cytoplasm. This balance is maintained by karyopherin‐mediated transport across the nuclear pore complex. Age‐associated dysfunction of karyopherins disrupts nucleocytoplasmic partitioning, leading to widespread proteostatic imbalance. Since distinct subcellular compartments provide unique environments for protein folding, stabilization, and degradation, mislocalization of proteins impairs multiple quality control systems simultaneously. Altered transport dynamics affect the behavior of biomolecular condensates, autophagic and proteasomal activities, chaperone function, and nucleolar homeostasis. Together, these changes promote protein crowding, aberrant phase transitions, and ribosomal dysfunction, ultimately driving the progressive collapse of proteostasis observed during aging.

Protein folding, degradation, assembly, and function occur in distinct subcellular environments with different crowding, redox states, and interaction landscapes (Labbadia and Morimoto 2015; Kaushik and Cuervo 2015). Indeed, proteostasis is inherently spatial and organelles manage proteins differently. For instance, while the nucleus provides a stabilizing environment, the cytoplasm exposes proteins to higher aggregation risk (Woerner et al. 2016). Maintaining this compartmental balance is therefore as important as folding or degradation capacity itself. The nuclear pore complex (NPC) is composed of hundreds of nucleoporins embedded in the nuclear envelope and enforces this balance by allowing passive diffusion of small molecules while requiring active transport for larger macromolecules. This selectivity is not static as NPC components are exceptionally long‐lived (Toyama and Hetzer 2013), accumulate damage over time, and are inefficiently replaced, rendering the pore especially vulnerable to aging. Even modest deterioration of NPC integrity alters permeability and transport dynamics, with disproportionate downstream consequences (Figure 1).

Karyopherins execute transport through a Ran GTPase–dependent cycle that continuously reshapes nuclear and cytoplasmic proteomes (Wing et al. 2022). Importantly, this process defines not only where proteins reside, but also how long they reside there. About a thousand cargoes cross each nuclear pore every second, highlighting cellular capacity for rapid and dynamic spatial modulation of the proteome. Karyopherin enrichment also prevents nuclear pore barrier dysfunction. Aging‐related changes in karyopherin abundance, affinity, or regulation therefore translate into altered residence times, aberrant accumulation, loss of temporal control over signaling pathways, as well as nuclear pore dysfunction. These phenotypes are generally observed in aged cells.

## 2 Karyopherins are Not Passive Carriers

A growing body of work has overturned the notion that karyopherins function solely as transport adaptors (Pasha et al. 2021). Several karyopherins directly bind disordered, aggregation‐prone regions of their cargos and act as molecular shields that suppress self‐association. In doing so, they regulate liquid–liquid phase separation and prevent pathological condensation. Dysfunction in this activity is at the center of aging phenotypes and neurodegeneration. Many proteins implicated in age‐associated diseases, including RNA‐binding proteins and transcriptional regulators, rely on karyopherins not just for localization but for solubility. When karyopherin availability is reduced or cargo recognition is impaired, these proteins undergo aberrant phase transitions that are difficult to reverse. Notably, this chaperone‐like function is progressively saturated during aging. Aged cells experience increased proteostatic load, transcriptional drift, altered post‐translational modifications, and chronic stress, all of which compete for limited karyopherin capacity. Thus, age‐related imbalance between cargo demand and karyopherin supply may be sufficient to tip the system toward aberrant aggregation. From this perspective, protein aggregation in aging is not simply a failure of folding; it is likely a failure of transport‐mediated buffering.

## 3 Aging as a Disorder of Nucleocytoplasmic Imbalance

Defective nucleocytoplasmic transport is now documented across diverse aging contexts (Springhower et al. 2020; Cagatay and Chook 2018), but it is rarely regarded as a driver. Age‐associated disruption of the Ran gradient, compromised NPC assembly, and altered karyopherin expression collectively impair proper and dynamic protein partitioning. Consequently, transport defects perturb membrane‐less organelles that depend on dynamic exchange with the nucleus and cytoplasm, including stress granules, nucleoli, and quality‐control compartments (QCC). The latter includes the Juxtanuclear QCC (JUNQ), the Intranuclear QCC (INQ), and the Insoluble Protein Deposit (IPOD), three critical intracellular sites for misfolded protein sequestration, refolding or eventual degradation (Kaganovich et al. 2008). As transport fidelity declines, these compartments become overloaded, less dynamic, and increasingly pathological.

The nucleolus, a membrane‐less organelle in the nucleus, illustrates how nucleocytoplasmic transport intersects with longevity. As the site of ribosomal subunit assembly, the size of the nucleolus is a strong indicator of cellular ribosomal biogenic capacity, and consequently the potential for cytoplasmic protein crowding. Nucleocytoplasmic transport controls the subcellular localization of ribosome subunits, but it also modulates ribosome biogenesis factors such as fibrillarin (Kumar et al. 2022). In collaboration with autophagy proteins, nucleocytoplasmic transport modulates nucleolar size and dynamics as well as ribosomal subunit levels, which in turn impacts ribosomal function, translational efficiencies, proteome specification, and ultimately longevity (Kumar et al. 2022). Reduced nucleolar size and activity correlate with lifespan extension in multiple organisms, consistent with the idea that attenuated and altered protein synthesis alleviates proteostatic pressure (Tiku et al. 2018). Transport‐mediated remodeling of nucleolar function may therefore act as a lever that couples aging to global proteome maintenance. Taken together, these observations suggest that aging can be viewed, in part, as a progressive failure to maintain nucleocytoplasmic asymmetry, where karyopherins are key players at fault.

## 4 Transcriptional Regulation via Karyopherins

Proper transcriptional output of longevity‐associated genes is generally modulated by the nucleocytoplasmic partitioning of transcription factors. Cells therefore employ karyopherins as gatekeepers of various transcriptional signatures, from growth to survival. In conjunction with gradual alterations in the epigenetic landscape, karyopherin dysfunction can exacerbate the transcriptional drift observed during aging by modulating the localization of key transcription factors and chromatin regulators. Notably, established longevity‐associated transcription factors (DAF‐16/FOXO and HLH‐30/TFEB) are readily subjected to post‐translational modifications that alter the dynamics of their import into and export out of the nucleus. Exportin 1 is an important modulator of the export of these transcription factors (Silvestrini et al. 2018), but its function also extends to chromatin dynamics and nuclear pore‐associated gene transcription (Ge et al. 2025). Altogether, karyopherin cargo‐specific modulations are reactive to growth, metabolic and proteostatic signals, and reinforce or attenuate stress response mechanisms elicited during aging. Therefore, appropriate transcriptional output requires the integration of signaling and karyopherin dynamics to properly position transcription factors.

## 5 Disease Contexts Reveal Dependence on Karyopherin Function

Many age‐related diseases display karyopherin dysfunction that impacts proteostasis, but also modify genomic mechanism and cellular states. Cancer cells actively rewire nucleocytoplasmic transport to sustain proliferation, evade checkpoints, and tolerate genomic instability. Exportin XPO1 exemplifies this strategy by exporting tumor suppressors and stress‐response factors from the nucleus, effectively neutralizing their function (Kumar et al. 2018). Aging enhances XPO1 levels which amplifies this vulnerability. Declining transport fidelity may lower the threshold for oncogenic transformation by weakening nuclear retention of protective factors and impairing DNA damage responses. Cancer thus reveals how transport asymmetry can be co‐opted to override cellular safeguards. In Hutchinson–Gilford progeria syndrome, disruption of the nuclear envelope catastrophically impairs nucleocytoplasmic transport, offering a compressed view of aging (Cisneros and Garcia‐Aguirre 2020). The striking overlap between progeroid phenotypes and physiological aging underscores the causal role of nucleocytoplasmic transport failure rather than positioning it as a downstream consequence. Neurodegenerative diseases provide another demonstration that nucleocytoplasmic transport is pathologically relevant. Alzheimer's disease, Parkinson's disease, Huntington's disease, ALS, and FTD all exhibit profound defects in nucleocytoplasmic partitioning (Pasha et al. 2021). Aggregation‐prone proteins that are normally regulated by karyopherins escape spatial control. Some karyopherins are misexpressed or sequestered into aggregates, exacerbating toxicity, while others act protectively by suppressing aggregation. This dichotomy argues against a generic transport collapse and instead points to selective vulnerability within the karyopherin network. From an aging perspective, these diseases may represent severe manifestations of a process that occurs more subtly in normal aging, that is, gradual erosion of karyopherin buffering capacity until phase behavior becomes pathological.

## 6 Targeting Karyopherins: From Cancer Therapy to Aging Intervention

The therapeutic success of targeting XPO1 in cancer has inadvertently validated nucleocytoplasmic transport as a druggable axis for other age‐related diseases. What is now becoming clear is that the benefits of modulating karyopherin activity extend well beyond oncology. In model organisms, partial inhibition of XPO1 restores nuclear localization of protective factors, enhances autophagy, and improves organismal healthspan (Kumar et al. 2022). XPO1 inhibition in ALS models enhanced survival (Silvestrini et al. 2018; Zhang et al. 2015), and it mediated improvement in spatial cognition in a murine AD model (Wong et al. 2026). Importantly, these benefits may arise not from shutting down transport, but from rebalancing it. This distinction is critical for aging applications, where subtle tuning rather than complete inhibition will be required. Importins are beginning to receive similar attention. Pharmacological or genetic attenuation of specific import pathways can block nuclear entry of oncogenic or aggregation‐prone proteins while sparing global transport. These findings argue that karyopherin dependence is context‐specific and therapeutically exploitable. The challenge moving forward is precision. Karyopherins handle many cargos, and indiscriminate inhibition is unlikely to be effective chronically. However, aging itself creates selective dependencies related to inflammatory signaling, stress adaptation, or proteostasis, which could represent therapeutic windows. Emerging strategies, including PROTACs, antisense oligonucleotides, and transient or tissue‐restricted interventions, offer promising routes to specificity.

## 7 Reframing Subcellular Protein Dynamics in Aging

The dominant narrative in proteostasis and aging research has focused on damage accumulation and declining repair (Figure 1). The work discussed here supports a complementary view: aging reflects loss of spatial regulation. Karyopherins, by coordinating transport, solubility, and phase behavior, are key to maintaining this regulation. In conjunction with other organelle‐to‐organelle and intra‐organelle trafficking, karyopherin dysfunction globally impairs proteostasis.

Several questions now require attention:

What defines karyopherin specificity in different proteostasis contexts?

How does nucleocytoplasmic transport regulate different biomolecular condensates?

Can enhancing karyopherin buffering delay proteostatic collapse without broadly perturbing nuclear–cytoplasmic exchange?

Is nuclear import an adaptive proteostasis pathway under stress?

How is karyopherin capacity allocated among competing cargos during aging?

Which transport routes become limiting first, and why?

How does altered nuclear transport drive inflammaging?

Are karyopherin dependencies disease‐ and tissue‐specific?

Can karyopherin activity be therapeutically tuned without global proteome and signaling interference?

How can spatial proteomic and transport profiling guide precision therapies?

Answering these questions will involve integrating nucleocytoplasmic transport into mainstream aging research rather than treating it as a specialized subfield. Doing so may reveal new principles of cellular organization and open avenues for interventions that restore proteostatic balance and help cells better manage the stress of aging.

## Author Contributions

Louis R. Lapierre: wrote the manuscript.

## Funding

L.R.L. is supported by grants from the Natural Sciences and Engineering Research Council of Canada (NSERC) and the Canadian Institutes of Health Research (CIHR) as well as a Research Chair in Precision Medicine from the J.‐Louis Lévesque Foundation.

## Conflicts of Interest

The author declares no conflicts of interest.

## Data Availability

Data sharing not applicable to this article as no datasets were generated or analysed during the current study.
